# An Anatomic Study on the Maxillary Sinus Mucosal Thickness and the Distance between the Maxillary Sinus Ostium and Sinus Floor for the Maxillary Sinus Augmentation

**DOI:** 10.3390/medicina56090470

**Published:** 2020-09-14

**Authors:** Il Hwan Lee, Do Hyun Kim, Soo Whan Kim, Jun-Beom Park, Sung Won Kim

**Affiliations:** 1 Department of Otolaryngology-Head and Neck Surgery, Seoul St. Mary’s Hospital, College of Medicine, The Catholic University of Korea, Seoul 02435, Korea; ilhwanloves@hanmail.net (I.H.L.); dohyuni9292@naver.com (D.H.K.); kshent@catholic.ac.kr (S.W.K.); 2 Department of Periodontics, College of Medicine, The Catholic University of Korea, Seoul 02435, Korea

**Keywords:** cone-beam computed tomography, dental implantation, maxillary sinusitis, risk factors

## Abstract

*Background and objectives:* The average rate of chronic sinusitis after maxillary implantation was approximately 5.1%. However, the evidence of predictive risk factors for sinusitis after implantation is lacking. The aim of this study was to perform an anatomic study on the maxillary sinus mucosal thickness (MSMT), the distance between the maxillary sinus ostium and sinus floor (MOD), and the MSMT/MOD ratio as a preoperative risk indicator for sinusitis after maxillary dental implantation. *Materials and Methods:* Between October 2008 and October 2019, all patients referred to the otolaryngology department were included in this study. A total of 120 patients were enrolled. The 95 patients who received no treatment prior to implantation were classified into Group A, the 16 patients who used antibiotics before implantation were classified into Group B, and the patients who had implants inserted after functional endoscopic sinus surgery were classified into Group C. The MSMT, MOD, MSMT/MOD ratio, anatomical factors associated with ostial obstruction, and the occurrence of postoperative sinusitis were reviewed. *Results:* There were significant group differences in MSMT (Group A vs. Group B, *p* = 0.001; Group B vs. Group C, *p* = 0.003; Group C vs. Group A, *p* < 0.0001). The MOD showed no significant difference among the three groups. The MSMT/MOD ratio showed significant differences between Groups A and B (*p* = 0.001), B and C (*p* < 0.0001), and C and A (*p* < 0.0001). Conclusions: It is important to check not only the proportion of the maxillary sinus occupying lesion, but also the status of the maxillary sinus osteomeatal complex when making therapeutic decisions. In addition, collaboration between dentists and otolaryngologists could improve outcomes in patients with maxillary sinus lesions.

## 1. Introduction

The use of dental implants has rapidly increased, and they are being used in almost all dentistry units in the country, resulting in a greater overall number of complications. In particular, low bone density and thin alveolar ridges can lead to various complications in the maxillary sinus [1]. The average rate of chronic sinusitis after maxillary implantation, among 25 studies, was approximately 5.1% [2]. Inflammatory edema in the maxillary sinus, membrane perforation, migration of graft material secondary to membrane perforation, and preexisting chronic sinusitis are known risk factors for postoperative sinusitis after dental implantation [2,3,4,5,6]. However, the risk factors are difficult to assess prior to implant placement in the maxillary sinus, apart from radiologically suspicious sinusitis. Some dentists have been exploring whether implants are appropriate in cases where abnormalities are detected in the maxillary sinus after panoramic radiography or cone beam computed tomography (CBCT). However, the evidence required to establish management guidelines after maxillary dental implantation is lacking. Therefore, we performed an anatomic study on the maxillary sinus mucosal thickness (MSMT), the distance between the maxillary sinus ostium and sinus floor (MOD), and the MSMT/MOD ratio as preoperative risk indicator for sinusitis after maxillary dental implantation, based on 11 years of experience of dentists and otolaryngologists at our hospital.

## 2. Materials and Methods

This study and the associated chart review were approved by the institutional review board of our hospital (approval no. KC19RESI0517). All patients referred from our periodontology department to the otolaryngology department, between October 2008 and October 2019 for dental implants, were included. Before the implant was placed, all patients had a CBCT scan taken. A total of 120 patients were enrolled in this study. Diabetes mellitus, asthma, past history of endoscopic sinus surgery, MSMT (including any cysts or solitary polyps), MOD, MSMT/MOD ratio, obstruction of the maxillary sinus ostium, anatomical factors potentially associated with ostial obstruction (such as paradoxical middle turbinate or Haller cells), and the occurrence of postoperative sinusitis were reviewed.

The patients were divided into three groups. The 95 patients who received no antibiotic treatment prior to implantation were classified into Group A, and the 16 patients who were using antibiotics before implantation were classified into Group B. The remaining nine patients, in whom implants were inserted after functional endoscopic sinus surgery (FESS), were classified into Group C.

All measured parameters are expressed as means ± standard deviation. A normality test was performed, and differences between two groups were analyzed by using Student’s *t*-test or the Mann–Whitney test. Pre- and post-treatment differences were analyzed by using the paired *t*-test or Wilcoxon signed rank test. Differences among the groups were analyzed by using the Kruskal–Wallis test. The post hoc Mann–Whitney test was applied, with the significance level established by using Bonferroni’s method. A *p*-value < 0.05 was considered to indicate statistical significance. All statistical analyses were conducted by using SAS software (ver. 9.4; SAS Institute, Cary, NC, USA).

## 3. Results

The mean patient age was 60.1 ± 13.5 years. There were 50 (47.6%) males and 55 (52.4%) females. Patients with diabetes mellitus (*n* = 16), asthma (*n* = 3), or a history of endoscopic sinus surgery (*n* = 4) were included. No postoperative complications were observed in these patients. A total of 120 maxillary sinus patients were examined, using CBCT. The mean MSMT (7.97 ± 8.47 mm), mean MOD (30.36 ± 5.51 mm), and mean MSMT/MOD ratio (0.26 ± 0.26) were obtained.

**Figure 1:**
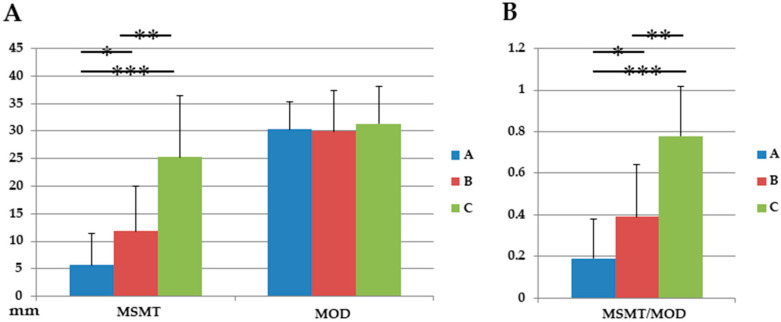
(**A**) Maxillary sinus parameters: comparison between the groups. Values for the maxillary sinus mucosal thickness (MSMT) and distance between the maxillary sinus ostium and sinus floor (MOD) (**A**) MSMT/MOD ratio (B). * *p* < 0.05 Group A vs. Group B; ** *p* < 0.05, Group B vs. Group C; *** *p* < 0.05, Group C vs. Group A.

The mean MSMT was 5.68 ± 5.74 mm in Group A, 11.84 ± 8.22 mm in Group B, and 25.3 ± 11.1 mm in Group C. There were significant differences between the groups (Group A vs. B, *p* = 0.001; Group B vs. Group C, *p* = 0.003; Group C vs. Group A, *p* < 0.0001). The mean MOD was measured to be 30.33 ± 5.08 mm in Group A, 29.98 ± 7.31 mm in Group B, and 31.35 ± 6.81 mm in Group C; there were no significant differences in the between-group comparisons (Group A vs. Group B, *p* = 0.804; Group B vs. Group C, *p* = 0.502; Group C vs. Group A, *p* = 0.637). The mean MSMT/MOD ratio was calculated to be 0.19 ± 0.19 in Group A, 0.39 ± 0.25 in Group B, and 0.78 ± 0.24 in Group C; there were significant differences between Groups A and B (*p* = 0.001), B and C (*p* < 0.0001), and C and A (*p* < 0.0001) (Figure 1).

Sinusitis after maxillary dental implantation was detected in 2 out of 120 cases. Details of the cases with sinusitis complications are provided in Table 1. Anatomical variations in the nasal cavity among the 120 cases were analyzed based on CBCT. Concha bullosa (*n* = 30, 25.0%), Haller cells (*n* = 13, 11.0%), and paradoxical curvature of the middle concha (*n* = 1; 0.8%) were seen in some cases. There were no anatomical variation-related cases of postoperative sinusitis. However, obstruction of the maxillary sinus ostium, which was another risk factor identified by CBCT, was observed in two cases (1.7%). In one case, which did not respond to antibiotics, the MSMT/MOD ratio was 0.6. Therefore, FESS, and implantation thereafter, was performed. In another case, the MSMT/MOD ratio was 0.23; therefore, we applied dental implants, but postoperative sinusitis nevertheless occurred in this case.

## 4. Discussion

There have been several reports on the relationship between maxillary sinus mucosal thickness and sinusitis after receiving maxillary dental implants [7,8]. Pneumatization of the maxillary sinuses, which shows variation among patients, affects the selection of implant methods. Pneumatization of the maxillary sinus in the edentulous maxillary ridge limits the volume of bone available for implant placement; maxillary sinus augmentation has been used to address this issue [9]. Maxillary sinus augmentation can be performed via a lateral or crestal approach [10,11]. Intraoperative complications associated with the lateral approach include sinus membrane perforation and bleeding, while postoperative complications include sinus graft infections, sinus infections, and sinusitis [12]. The following methods can be used to reduce or minimize complications in the posterior maxilla. With the crestal approach, which is considered less invasive, the application of hydraulic pressure may decrease perforations and increase survival rates [10]. It was previously believed that at least 5 mm of bone below the maxillary sinuses was required for application of the crestal approach [12]. However, in a more recent report, the crestal approach was applied with only 2 mm of alveolar bone available [13]. Sinus augmentation has been performed without graft materials, and a previous study reported no significant difference in short-term implant survival in maxillary sinus augmentation with versus without grafts [14]. The application of short implants may minimize complications. Previous reports compared outcomes between ≤ 6-mm- and ≥10-mm-long implants, placed after both lateral and transcrestal sinus augmentation; it was suggested that placement of short implants may be a more reliable option [15]. With the use of these methods, the success rates of maxillary sinus augmentation and maxillary dental implantation have improved.

However, the risk factors associated with the maxillary sinusitis have not been evaluated thoroughly. Ostial obstruction is believed to be one such risk factor, and it is important to both check for maxillary sinus pneumatization and measure the thickness of the mucous membrane. Chen et al. suggested that, when the height of polyp, cyst, or mucosal thickening was not less than half of maxillary sinus, treatment of sinusitis and preventive FESS was needed [16]. Chan et al. reported that it is necessary to consult with an otolaryngologist if mucosal thickening of maxillary sinus is more than one-third [17]. However, there is a lack of clinical evidence supporting this assertion. Therefore, in this study, we conducted this pilot study and explained the necessity to derive the related evidences.

Only 1.6% of the chronic sinusitis cases after maxillary implant were referred, compared to average rate of 5.1% for other 25 studies in this study. We thought that it is because of close consultation between dentists and otolaryngologists. In all the cases where any lesion in the maxillary sinus is identified in preoperative CBCT, dentists refer the patient to an otolaryngologist for assessment. Thus, the occurrence of complications may be lowered by reducing the risk factors before implants. Moreover, FESS is used if antibiotics confer no therapeutic benefits and the risk of developing chronic sinusitis after implantation is considered high. Our institute appears more strict with respect to the application of surgical procedures (mean MSMT/MOD ratio: 0.78 ± 0.24), compared to previous reports [16,17]. However, one case with ostium obstruction showed postoperative sinus complications, although the MSMT/MOD ratio was low (0.23). Based on these results, not only the MSMT and MSMT/MOD ratio, but also osteomeatal status, may be associated with postoperative sinusitis complications after dental implants. Therefore, for patients with maxillary sinus lesions who are unresponsive to antibiotics, FESS may be appropriate depending on the osteomeatal status and MSMT/MOD ratio. Previous studies have focused mainly on the state of the maxillary sinus floor regarding maxillary implants. Therefore, the key point of this manuscript is that the relative occupying lesion such as mucosal thickening or a cyst or solitary polyp should be assessed with maxillary sinus ostium position and the depth of maxillary sinus. Through this point, it was also to be discussed that, when taking cone-beam-computed tomography and imaging, and evaluating the entire maxillary sinus, including the maxillary sinus ostium, it would be an important criterion for evaluating surgery.

There were some limitations to this study. First, its retrospective nature limits the power of the results, compared to randomized controlled studies. Second, we could not perform routine postoperative CT in all enrolled patients, in consideration of the risk–benefit ratio of radiation exposure and cost of the examination. Third, all patients were seen at a single institute, i.e., our tertiary medical center, so selection bias is possible. Nevertheless, this study, which was characterized by close collaboration between two departments to confirm each patient’s prognosis, provides important data that could facilitate future assessments of dental-implantation patients.

## 5. Conclusions

In this study, MSMT, MOD, MSMT/MOD ratio, obstruction of the maxillary sinus ostium, anatomical factors potentially associated with ostial obstruction, and past history of endoscopic sinus surgery were analyzed, and the results showed that significant group differences were noted in MSMT and MSMT/MOD ratio. It can be suggested that it is important to evaluate the status of the maxillary sinus complex during therapeutic decision-making prior to maxillary dental implantation, to decrease the likelihood of postoperative sinusitis. In addition, collaboration between dentists and otolaryngologists could improve outcomes in patients with maxillary sinus lesions.

## Figures and Tables

**Table 1:** Case-based analysis of patients experiencing unilateral maxillary sinusitis complications after dental implants.

Patient Number	Group	Sex	Age (years)	Sinus Side (Tooth Number)	Anatomical Variation in OMU	MOD (mm)	MSMT (mm)	MSMT/MOD Ratio	Other Risk Factors on CBCT	Medical Treatment	Surgical Treatment
1	B	Male	85	Right (16)	None	37.2	18	0.48	None	Yes	None
2	A	Male	55	Left (26)	None	33	7.5	0.23	Ostial obstruction	Yes	Yes

OMU, ostiomeatal unit; MOD, the distance between the maxillary sinus ostium and sinus floor; and MSMT, the maxillary sinus mucosal thickness.
